# Detecting and Removing Ascertainment Bias in Microsatellites from the HGDP-CEPH Panel

**DOI:** 10.1534/g3.111.001016

**Published:** 2011-11-01

**Authors:** Anders Eriksson, Andrea Manica

**Affiliations:** Evolutionary Ecology Group, Department of Zoology, University of Cambridge, Cambridge, CB2 3EJ, United Kingdom

**Keywords:** microsatellites, ascertainment bias, HGDP-CEPH, rarefaction

## Abstract

Although ascertainment bias in single nucleotide polymorphisms is a well-known problem, it is generally accepted that microsatellites have mutation rates too high for bias to be a concern. Here, we analyze in detail the large set of microsatellites typed for the Human Genetic Diversity Panel (HGDP)-CEPH panel. We develop a novel framework based on rarefaction to compare heterozygosity across markers with different mutation rates. We find that, whereas di- and tri-nucleotides show similar patterns of within- and between-population heterozygosity, tetra-nucleotides are inconsistent with the other two motifs. In addition, di- and tri-nucleotides are consistent with 16 unbiased tetra-nucleotide markers, whereas the HPGP-CEPH tetra-nucleotides are significantly different. This discrepancy is due to the HGDP-CEPH tetra-nucleotides being too homogeneous across Eurasia, even after their slower mutation rate is taken into account by rarefying the other markers. The most likely explanation for this pattern is ascertainment bias. We strongly advocate the exclusion of tetra-nucleotides from future population genetics analysis of this dataset, and we argue that other microsatellite datasets should be investigated for the presence of bias using the approach outlined in this article.

The rapidly decreasing cost of high-throughput genotyping means that large datasets with both detailed genetic coverage and wide geographic scope are finally a reality (*e.g.*, Li *et al.* 2008; Novembre *et al.* 2008; Jakobsson *et al.* 2008). These datasets provide fantastic opportunities to investigate human historical demography, and they promise to allow us to unravel the relative role of mutation, drift, and selection in shaping human diversity. However, the sets of single nucleotide polymorphisms (SNP) typed by current technologies were originally chosen to represent diversity within panels of narrow geographic scope (Tishkoff and Verrelli 2003; Carlson *et al.* 2003) and thus suffer strongly of ascertainment bias (Rogers and Jorde 1996; Kuhner *et al.* 2000; Wakeley *et al.* 2001; Akey *et al.* 2003; Bustamante *et al.* 2005), making their use problematic when reconstructing the past of human populations at a worldwide level.

A key step in assessing the importance of ascertainment bias in affecting demographic reconstructions has been the comparison with similar analyses performed on microsatellites (also known as single-tandem repeats, STR). Ascertainment schemes have been argued to have little effect on these highly polymorphic markers (Bowcock *et al.* 1994; Rogers and Jorde 1996; Harpending and Rogers 2000), which are generally thought to provide an unbiased estimate of neutral variation; thus, they are used as a yardstick against which attempts to remove ascertainment bias from SNP are assessed. For example, when analyzing a dataset of 3024 SNPs from the Human Genetic Diversity Panel [HGDP-CEPH (Cann *et al.* 2002), arguably the most comprehensive dataset on human genetic diversity covering over 1000 individuals from 52 populations], Conrad *et al.* (2006) investigated how using differently sized windows to define haplotypes affected estimates of within-population heterozygosity and concluded that a window of 20 Kb provided the best estimates as it gave the tightest fit with similar estimates obtained from 783 STRs typed in the same populations. Later analyses of a larger number of SNPs from the same panel have kept comparing the STR data, validating a variety of approaches, such as investigations of isolation by distance using patterns of between-population diversity (*F*_ST_) (Jakobsson *et al.* 2008), of decrease in within-population variability with increasing distance from Africa (Li *et al.* 2008), and of population clustering using STRUCTURE (Li *et al.* 2008; Jakobsson *et al.* 2008).

The assumption that microsatellites are free of bias has been questioned by two studies looking at the HGDP-CEPH data (Ray *et al.* 2005; Foll and Gaggiotti 2006). Romero *et al.* (2009) failed to find a bias when comparing the full set of 783 STRs used in the HGDP-CEPH dataset with a set of 16 unbiased STRs discovered in a multiethnic panel; however, the small number of markers in the unbiased set limits the power of their analysis. Here, we look in detail for signs of ascertainment bias in the 783 STRs used in the HGDP-CEPH panel (Rosenberg 2006). Specifically, we compare results obtained using different statistical approaches applied to di-, tri-, and tetra-nucleotides separately, as we would expect the effect of bias to depend on the different mutation rates found for markers with different motif lengths. As some inconsistencies in results using different motifs might be ascribed to their different sensitivity to demographic processes (such as bottlenecks) rather than ascertainment, we develop a metric that reflects the underlying gene genealogy, effectively providing a description of diversity that is unaffected by mutation rates. More specifically, we use a rarefaction framework to generate estimates of expected heterozygosity (both within and between populations) that are rescaled to a single reference mutation rate and, thus, are directly comparable from a statistical point of view. Using this novel framework, which removes the differential effect of demographic processes on markers with different mutation rates, we demonstrate that tetra-nucleotides are inconsistent with di- and tri-nucleotides, a likely sign of ascertainment bias.

## Materials and Methods

### Identifying and removing inconsistencies in STR genotype data

We first developed a simple approach to determine the motif length of microsatellite markers from their fragment lengths, providing an objective way of removing inconsistencies from the data prior to our analysis. All fragment lengths *L* at a given locus can be written in the form *L* = *L_m_ n* + γ, where *L_m_* is the length of the locus’s repeat unit, *n* is the number of repeat units (plus an unknown offset from the flanking regions), and γ is an integer between zero and *L_m_* − 1 (the remainder of dividing *L* by *L_m_*). For each locus, we are interested in obtaining a reliable estimate of *L_m_* and γ.

The analysis, which is repeated for each locus, consists of three steps. First, *L*_m_ is determined by finding the shift (by 2, 3, 4, or 5 nucleotides) that maximizes the relative overlap $\sum_{i}f_{i}f_{i+L_{m}}/\sum_{i}f_{i}^{2}$, where *f_i_* is the frequency of allele *i* in the sample. Second, we calculate the remainder γ from dividing each allele length *L* by *L_m_*. Ideally, there should be a unique value of γ obtained from all lengths *L* for a given locus. In reality, several loci have multiple values. Loci for which the most common value of γ exceeded 95% of the total count were cleaned by recoding individuals with at least one allele with an unusual offset as missing data for that locus (supporting information, Figure S1, A and B); loci with less than 95% estimates of γ with same value were excluded from future analysis as they deviate too strongly from the assumed model (Figure S1, C and D). For all clean loci, allele lengths were converted into repeat numbers as *n* = (*L* − γ)/*L_m_*. A Matlab implementation of this procedure is available in File S1.

### Statistical analysis of filtered genotype data

We investigated the results obtained for different motif lengths from two types of statistical approaches: hierarchical clustering with STRUCTURE v. 2.2.3, (Pritchard *et al.* 2000) and pairwise population differentiation estimated as *F*_ST_ [calculated in Matlab v7.11 from the expected within- and between-population heterozygosities weighted according to sample size (Nei 1978)]. We would expect both clustering and *F*_ST_ patterns to be relatively robust to differences in mutation rates between the markers with different motif lengths (Haasl and Payseur 2011).

### Mutation rarefaction

Demographic processes such as bottlenecks might be sampled differently by markers with different mutation rates. Even the normalization of between- by within-population variability used when computing pairwise *F*_ST_ will not fully account for this effect. To be able to statistically compare markers with different mutation rates, we would ideally need a metric that reflects the underlying gene genealogy, effectively providing a description of diversity that is unaffected by mutation rates. To this end, we have developed a rarefaction framework to generate estimates of expected heterozygosity (both within and between populations) that are rescaled to a single reference mutation rate and, thus, are directly comparable from a statistical point of view.

Heterozygosity, defined as the probability that two alleles sampled at random are identical, is a frequently used measure of genetic diversity. It is most commonly computed to estimate within-population diversity, but it can be equally applied to describe between-population differentiation. However, heterozygosity estimates from markers with different mutation rates cannot be compared directly and do not obey a simple scaling rule; how heterozygosity scales with mutation rate depends on the detailed shape of the underlying gene genealogy (and thus on the distribution of time to most recent common ancestor, TMRCA). To solve this problem, we analyze the more general question of how the distribution *p*(Δ) of pairwise differences in repeat count, Δ, depends on the mutation rate.

**Figure 1:**
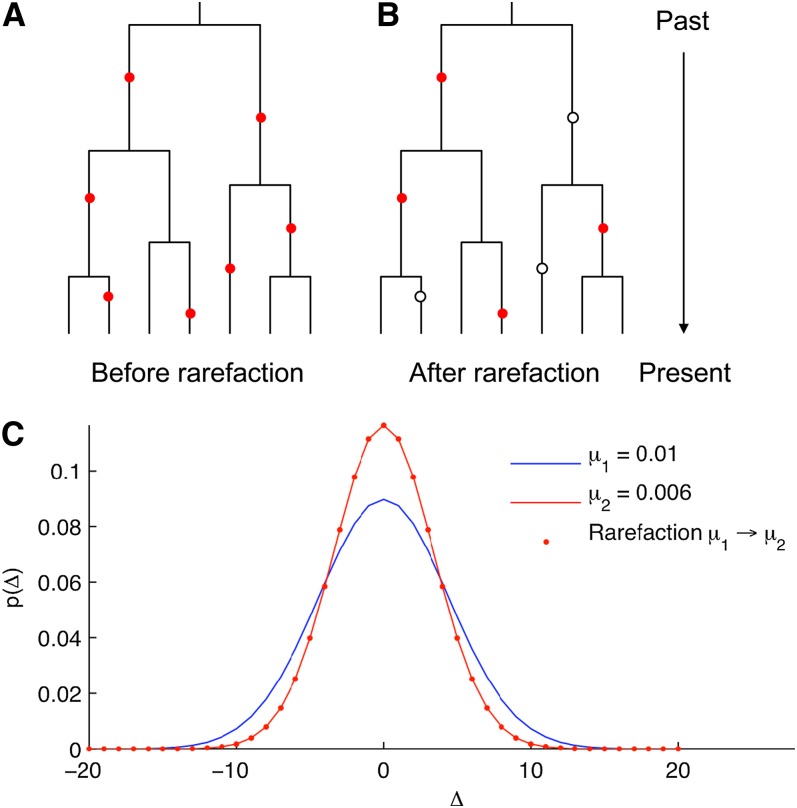
Illustration of the mutation rarefaction principle for a given locus. (A) The gene genealogy of the sample (solid lines) and the location of the mutations since the most recent common ancestor (red circles). (B) Randomly removing a fraction of the mutations (open circles) tends to reduce the heterozygosity observed the sample. However, the extent to which this happens depends on the location of the deleted mutations in the gene genealogy. (C) The effect of rarefaction on the distribution of difference *D* in repeat count between individuals, *p*(*D*), with MRCA 1000 generations ago. The blue and red curves show the distributions for mutation rates *m*_1_ = 0.01 and *m*_2_ = 0.006, respectively, and the red dots show the result of rarefaction of the blue distribution by a factor *m*_2_/*m*_1_ = 0.6 (using Equation 2).

The principle of our method is best understood by considering a single microsatellite locus with mutation rate μ. What would *p*(Δ) be if we had a lower mutation rate, μ′? If we knew the underlying gene genealogy of locus in a sample, and the location of mutations on the gene genealogy, we could rarefy the mutations to the new mutation rate by randomly removing mutations such that on average a fraction μ′/μ of the mutations remains, and calculate the new *p*(Δ) from the resulting genetic variation. This principle is illustrated in Figure 1, A and B, and the effect of mutation rarefaction on the distribution *p*(Δ) is illustrated in Figure 1C.

In reality, we can observe p(Δ), but we do not know the underlying gene genealogy, how many mutations occurred, or where they are placed on the gene genealogy. Nevertheless, for large samples, it is possible to estimate the rarefied *p*(Δ) from the observed distribution by implicitly removing a given fraction of the underlying mutations. Consider the characteristic function of the distribution *p*(Δ), defined as

$$\hat{p}(\omega )=\sum_{\Delta }p(\Delta )\cos(\Delta \omega )$$

Using the standard Stepwise Mutation Model [SMM, Kimura and Ohta (1975, 1978)], we can express this function in terms of the (unknown) distribution of TMRCA for pairs of individuals. In the SMM, each mutation leads to the addition or removal of single repeat units, with equal probability. Assuming a mutation occurs independently with rate μ per generation, the difference Δ in repeat count for a given pair of individuals with *t* generations to the MRCA can be written as the sum of 2*t* independent random variables, which are −1, 0, or 1 with probability μ/2, 1 − μ, or μ/2, respectively. As the characteristic function of the sum of two independent variables is the product of their characteristic functions, and each variable here has the characteristic function 1−μ+μcosω, the characteristic function of Δ is ${\left(1-\mu +\mu \cos\omega \right)}^{2t}$. For a sample of more than two individuals, we average the characteristic function over the distribution *g_t_* of pairwise time to MRCA in the sample:

$$\hat{p}(\omega ,\mu )=\sum_{t=0}^{\infty }g_{t}{\left(1-\mu +\mu \cos\omega \right)}^{2t}=\tilde{g}\left(1-\mu +\mu \cos\omega \right)$$

where $\tilde{g}\left(z\right)=\sum_{t=0}^{\infty }g_{t}z^{2t}$ is a generating function for *g_t_*. Solving for ω as a function of *z*, one obtains

$$\tilde{g}\left(z\right)=\hat{p}\left(\arccos\left[1-\frac{1-z}{\mu }\right],\mu \right)$$

As the underlying distribution of time to the MRCA is assumed to be independent of the mutation rate (because of neutrality), this expression holds for any mutation rate. Hence, for mutation rate μ′, we have

$$\hat{p}(\omega ,\mu \prime )=\tilde{g}\left(1-\mu \prime +\mu \prime \cos\omega \right)=\hat{p}\left(\arccos\;\left[1-\frac{\mu \prime }{\mu }\left(1-\cos\omega \right)\right],\mu \right)$$

Writing $\hat{p}(\omega ,\mu )$ in terms of Δ gives an explicit expression for the rarefied distribution of Δ in terms of the original distribution:

$${\hat{p}}_{\text{rarefied}}(\omega )=\sum_{\Delta }p(\Delta )\cos\left(\Delta \arccos\;\left[1-\frac{\mu \prime }{\mu }\left(1-\cos\omega \right)\right]\right)$$ (1)

From the characteristic function, we can obtain the probability of observing difference Δ in repeat counts between individuals, *p*_rarefied_(Δ), in the rarefied sample by taking an inverse Fourier transform:

$$p_{\text{rarefied}}(\Delta )=\sum_{\Delta \prime }p(\Delta \prime )\frac{1}{\pi }\int_{0}^{\pi }\cos(\Delta \omega )\cos\;\left(\Delta \prime \arccos\;\left[1-\frac{\mu \prime }{\mu }\left(1-\cos\omega \right)\right]\right)d\omega$$ (2)

Figure 1C illustrates the effect of rarefaction on *p*(Δ). Finally, using this relation, we obtain the following scaling rule for heterozygosity:

$$H_{\text{rarefied}}=1-p_{\text{rarefied}}(0)$$

Mathematically, this method only works when going from larger to smaller mutation rates. This makes intuitive sense, because for μ′ < μ, the method is removing information, but going in the opposite direction would correspond to increasing the amount of information in the sample. This type of extrapolation is so unstable that it is useless for all practical purposes.

Finally, as a sanity check, we verify that our scaling method obey the scaling relation $V_{\text{rarefied}}=(\mu \prime /\mu )V$, where *V* is the variance of the counts of repeat units, which is the standard method in SMM theory for calibrating the relative mutation rate of two markers [*e.g.*, see Zhivotovsky *et al.* (2003) and references therein]. It is straightforward to show that this relation holds for the rarefaction method, using the Taylor expansion of Equation 1 around ω = 0.

### Estimating mutation rates from mutation rarefaction

For the markers used in the HGDP-CEPH panel, only the mutation rate for di-nucleotides has been estimated directly from pedigree data [μ_2_ = 1.52 × 10^−3^, Zhivotovsky *et al.* (2000)], whereas mutation rates for tri- and tetra-nucleotides have to be estimated indirectly (we do not consider penta-nucleotides, as there are too few of them). As the scaling rule for heterozygosity depends only on the ratio of mutation rates (*i.e.*, on the fraction of mutations kept in the rarefaction process), we can use it to find the best estimates of mutation rates in tri- and tetra- nucleotides.

For example, if we want to estimate the mutation rate for tri-nucleotides, we can find the optimal rarefaction factor, *k*_23_ (*i.e.*, μ_3_/μ_2_), that brings the within- and between-population heterozygosities of di-nucleotides closest to those of tri-nucleotides. The mutation rate estimate of tri-nucleotides is then μ_3_ = *k*_23_μ_2_. An additional estimate for tri-nucleotides can be obtained using tetra-nucleotides as a reference for scaling the mutation rate of di- and tri-nucleotides. Given the optimal rarefaction factors *k*_24_ (*i.e.*, μ_4_/μ_2_) and *k*_34_ (*i.e.*, μ_4_/μ_3_), we estimate the mutation rate of tri-nucleotides as μ_3_ = μ_2_*k*_24_/*k*_34_.

### Trends in within- and between-population heterozygosity

To test whether the matrix of within- and between population heterozygosities of motifs with different lengths are consistent after scaling, we separately scaled di- and tri-nucleotide markers to match the HGDP-CEPH tetra-nucleotide markers (by minimizing mean-square difference of the heterozygosity matrices). To test whether the one-to-one relation can be significantly rejected, we estimated 95% confidence intervals of the regression lines (di- and tri-nucleotide heterozygosities *versus* tetra-nucleotide heterozygosities) from 10,000 bootstrap samples of the data using a Matlab script. We then repeated the same analysis comparing di-, tri- and tetra-nucleotide heterozygosities to the heterozygosities of the unbiased tetra-nucleotide markers, after scaling to this set of markers.

Finally, we analyzed the dependence of within-population expected heterozygosity and allelic richness [calculated using ADZE v.1.0 (Szpiech *et al.* 2008)] on the distance from sub-Saharan Africa [calculated as in (Manica *et al.* 2005)] for di-, tri-, and tetra-nucleotide markers. Especially, we analyzed the effect of subsetting the markers and scaling of heterozygosities on the cline in heterozygosity away from sub-Saharan Africa.

## Results

### Identifying and removing inconsistencies in STR genotype data

Despite our strict demands for accepting a locus, our approach gave 749 markers (out of 783) that could be used for analysis, subdivided into 54 di-, 166 tri-, 519 tetra-, and 10 penta-nucleotides (Table S1). The 10 penta-nucleotide markers were dropped from further analysis because they were deemed too few. The cleaned data from the 749 markers [as well a cleaned version of the 16 unbiased markers from Romero *et al.* (2009)] are available (File S1 and File S2). For those markers that had been previously classified based on the reference Human Genome sequence (Pemberton *et al.* 2009), Pemberton *et al.* and our classifications were in agreement, with the exception of five markers (Table S2, File S3, and File S4).

### The effect of motif length on STRUCTURE clusters and *F*_ST_ patterns

**Figure 2:**
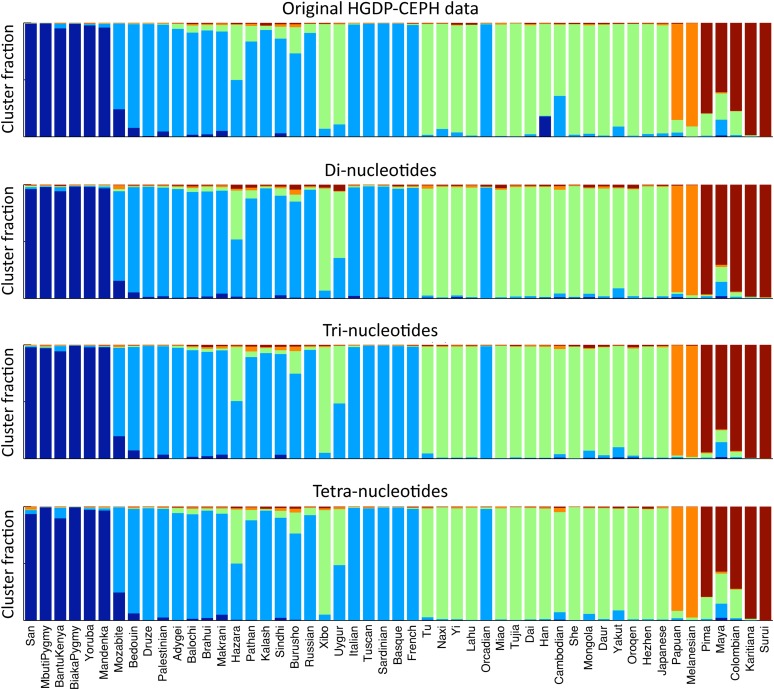
Five-cluster STRUCTURE analysis of the HGDP-CEPH data, with populations ordered according to increasing distance from sub-Saharan Africa. The top panel shows the clusters of the original HGDP-CEPH dataset. The remaining panels show, from top to bottom, the respective clusters based on the di-, tri-, and tetra-nucleotides in the cleaned HGDP-CEPH dataset.

**Figure 3:**
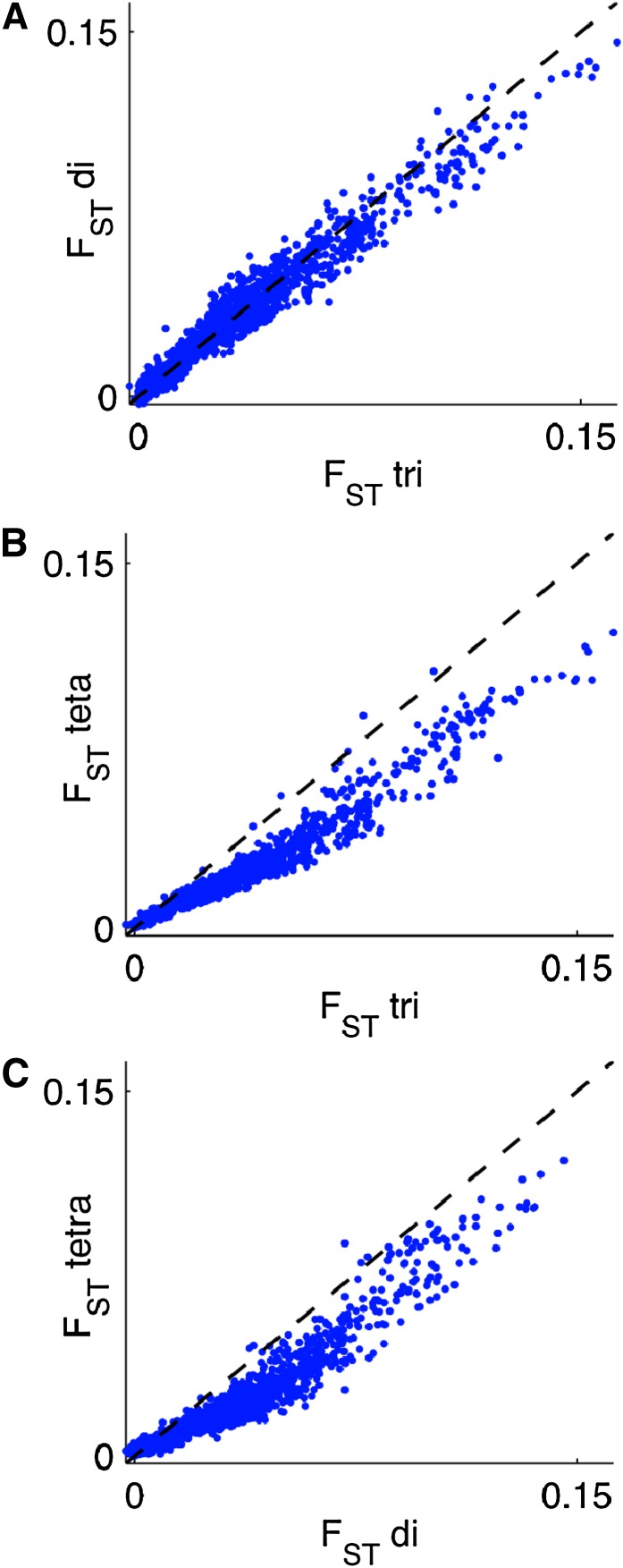
*F*_ST_ of cleaned HGDP-CEPH markers with different repeat motif lengths. The three panels show pairwise comparisons between *F*_ST_ values: (A) di- *versus* tri-nucleotides, (B) tri- *versus* tetra-nucleotides, and (C) di- *versus* tetra-nucleotides.

Analysis of di-, tri- and tetra-nucleotides with STRUCTURE yielded the same clusters irrespective of which motif length was used (Figure 2). However, when comparing *F*_ST_ patterns for these three categories of markers, we found a strong effect of marker length for *F*_ST_ patterns: di- and tri-nucleotides were in very good correspondence (Figure 3, A and B), but tetra-nucleotides showed much lower population differentiation than the shorter motifs (Figure 3C). To determine whether this discrepancy can be attributed to demographic processes being sampled differently by markers with dissimilar mutation rates, we apply our rarefaction approach to compute statistically comparable, rarefied heterozygosity estimates.

### Mutation rates from mutation rarefaction

To rarefy estimates of within- and between-population expected heterozygosity, we first use our scaling procedure to find the relative mutation rates of the different groups of markers (see *Materials and Methods*). Table 1 shows the result of estimating the mutation rates of di-, tri-, and tetra-nucleotides, as well as a set of 16 unbiased tetra-nucleotides from Romero *et al.* (2009). Each row corresponds to using a given reference set of markers, and the columns contain the mutation estimates for different sets. In addition to fitting the within- and between-population heterozygosities, *p*(0), we show mutation rates calculated by fitting the distribution of count differences *p*(Δ) and by scaling the variances (the approach used in previous articles). We find that the mutation rates obtained by rarefaction analysis are insensitive to which markers are used as reference and whether the fitting was based on the full distribution of differences, *p*(Δ), or on only the heterozygosities, *p*(0). The scaling of variance, however, yields a slightly lower mutation rate for the tri-nucleotide loci (0.65 × 10^−3^ *versus* 0.71 × 10^−3^ – 0.77 × 10^−3^). There is also a mismatch for the mutation rate for the unbiased tetra-nucleotides (0.46 × 10^−3^ *versus* 0.23 × 10^−3^), but the small number of markers makes these estimates somewhat unreliable. For comparison, we also show the mutation rates found by Zhivotovsky *et al.* (2003), which are based on a subset of the CEPH-HGDP markers obtained by removing markers with extreme variances.

**Table 1:** The mutation rates of di-, tri- and tetra-nucleotide markers in the cleaned data and the unbiased tetra-nucleotide markers, when scaling down to different reference markers

	Reference	Di*^a^*	Tri	Tetra	Unbiased Tetra
Fitting full distribution	Tri	1.52 × 10^−3^	0.77 × 10^−3^	–	–
	Tetra	1.52 × 10^−3^	0.74 × 10^−3^	0.58 × 10^−3^	–
	Unbiased	1.52 × 10^−3^	0.76 × 10^−3^	0.56 × 10^−3^	0.23 × 10^−3^
Fitting heterozygosity	Tri	1.52 × 10^−3^	0.71 × 10^−3^	–	–
	Tetra	1.52 × 10^−3^	0.73 × 10^−3^	–	–
	Unbiased	1.52 × 10^−3^	0.76 × 10^−3^	0.56 × 10^−3^	0.23 × 10^−3^
Fitting variance		1.52 × 10^−3^	0.65 × 10^−3^	0.52 × 10^−3^	0.46 × 10^−3^
Zhivotovsky *et al.*		1.52 × 10^−3^	0.71 × 10^−3^	0.64 × 10^−3^	–

The markers were fitted using the full distribution of difference in allele length (upper section), using only the heterozygosity (middle section), and scaling of average variance of allele length (lower section). Dashes imply that the scaling could not be performed because the reference markers were more diverse than the scaled markers.

a Data from Zhivotovsky *et al.* (2000).

### Trends in within- and between-population heterozygosity

**Figure 4:**
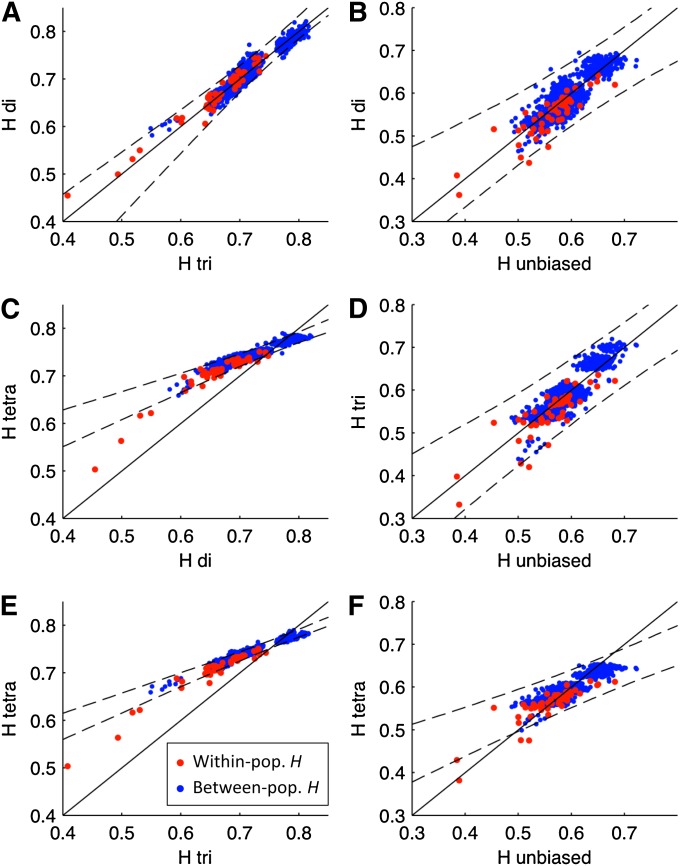
Pairwise comparisons of the scaled within- and between-population expected heterozygosities (red and blue dots, respectively). HGDP-CEPH markers are scaled to match the tetra-nucleotide markers (A, C, E) or unbiased tetra-nucleotide markers (B, D, F). In all panels, the solid black lines show the one-to-one relations, and the dashed black lines show the 95% confidence intervals of regression lines based on 10,000 bootstrap samples of the data. A, C, E: Comparisons of heterozygosities of di-, tri-, and tetra-nucleotide markers. B, D, F: comparisons of di-, tri- and tetra-nucleotide to the unbiased markers.

Figure 4 shows pairwise comparisons of the rarefied within- and between-population average heterozygosities of HGDP-CEPH markers after fitting to the tetra-nucleotide markers (left panels) or unbiased tetra-nucleotide markers (right panels, together with lines indicating the one-to-one relationships and 95% confidence intervals of the regression lines. The di-, tri- and unbiased tetra-nucleotides are in good agreement (Figure 4, A, B, and D), whereas the HGDP-CEPH tetra-nucleotides are significantly different from all other groups (Figure 4, C, E, and F), consistent with patterns in *F*_ST_ (Figure 3).

**Figure 5:**
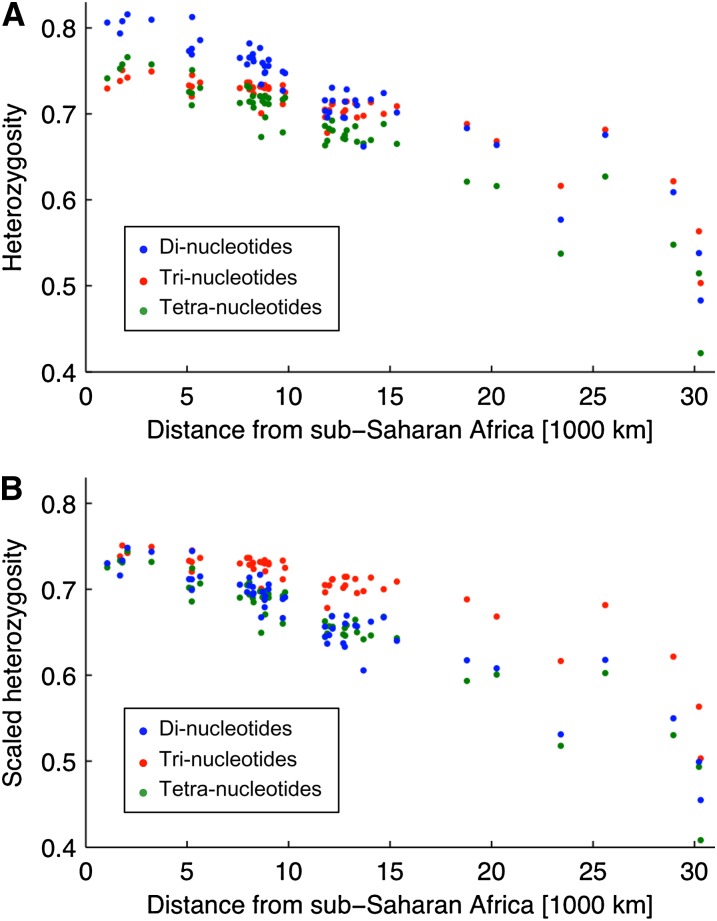
Within-population expected heterozygosity as a function of distance from sub-Saharan Africa for di-, tri-, and tetra-nucleotides separately (blue, green, red symbols, respectively). Panels A and B show the heterozygosity before and after scaling to match the full tetra-nucleotide *H*-matrix, respectively (see Table 1 for the relative mutation rates).

This discrepancy seems to be due to populations in Eurasia being relatively homogeneous for tetra-nucleotides. This effect can be visualized by plotting the decline in within-population genetic diversity with increasing distance from sub-Saharan Africa [a pattern attributed to the effect of sequential founder events during the spread out of Africa by anatomically modern humans (Prugnolle *et al.* 2005; Ramachandran *et al.* 2005; Manica *et al.* 2007)]. Irrespective of whether we look at raw estimates of expected within-population heterozygosity (Figure 5A) or at rarefied estimates using the approach outlined in this article (Figure 5B), it is clear that, whereas di- and tri-nucleotides show a smooth decline of heterozygosity with increasing distance from Africa, the heterozygosity of tetra-nucleotides is much flatter across Eurasia (corresponding to approximately 5,000–15,000 km in Figure 5), with a steep decline evident only once we reach the Americas. A similar pattern holds for allelic richness (Figure S2).

**Figure 6:**
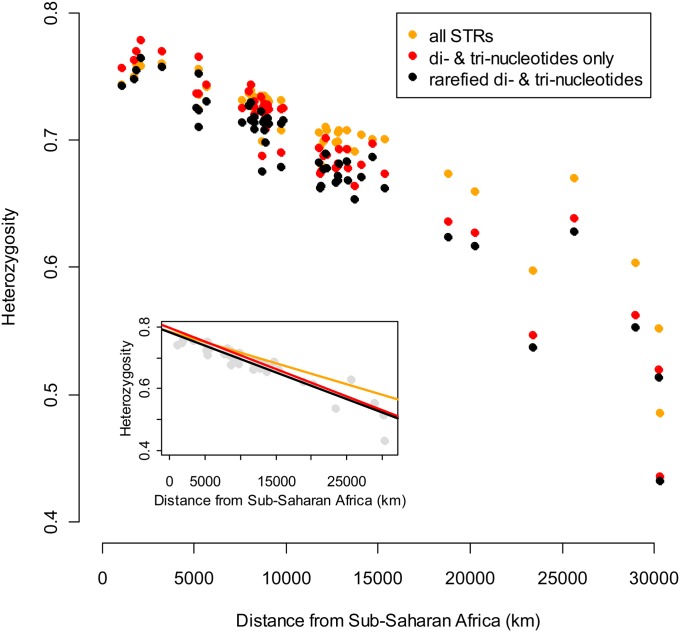
Average within-population heterozygosity for the CEPH-HGDP populations, as a function of distance from sub-Saharan Africa. Orange: Average of all cleaned markers. Red: Average of di- and tri-nucleotides. Black: Average of di- and tri-nucleotides, with di-nucleotides scaled down to the tri-nucleotides. Inset: Regression lines for the three colors. For reference, the scaled data are shown as gray.

To what extend do tetra-nucleotides affect previous estimates of relationship between within-population heterozygosity and distance from sub-Saharan Africa? To answer this question, we estimated the relationship for three different averages: (*i*) of the complete set of markers for the HGDP-CEPH panel (*i.e.*, pooling all di-, tri- and tetra-nucleotides and ignoring differences in mutation rates, as is commonly done when estimating within-population heterozygosity); (*ii*) of only the subset of di- and tri-nucleotides (*i.e.*, pooling only the markers that were consistent with each other, again ignoring mutation rates); and (*iii*) of an estimate of the latter after rarefying the di-nucleotide to the mutation rate of tri-nucleotides (*i.e.*, the correct way of combining markers with different mutation rates). As can be seen in Figure 6, the slope estimated for the full set of markers (−6.792·10^−6^ ± 4.277·10^−7^) is significantly shallower than that estimated for the subset of di- and tri-nucleotides (−8.866·10^−6^ ± 4.584·10^−7^; F_1,98_ = 10.94, *P* = 0.001) and for the rarefied dataset (−8.656·10^−6^ ± 4.498·10^−7^; F_1,98_ = 9.02, *P* = 0.003). The is no difference in the slope estimates for subsets of di- and tri-nucleotides and for the rarefied dataset (F_1,98_ = 0.11, *P* = 0.744), but as one would expect, the intercept is significantly lower for the latter (7.820·10^−01^ ± 5.929·10^−3^ *versus* 7.958·10^−1^ ± 6.043·10^−3^; F_1,99_ = 6.92, *P* = 0.010).

## Discussion

Our analyses clearly illustrates that, whereas di- and tri-nucleotides show patterns that are highly consistent with each other, the tetra-nucleotides originally typed for the HGDP-CEPH panel are inconsistent as they are too homogeneous in their individual distributions across Eurasia. This discrepancy cannot have arisen from demographic processes affecting markers with different mutation rates to different extents: rarefying diversity to the slowest mutation rate should always give equivalent estimates, as the underlying distribution of gene genealogies is the same irrespective of the marker of choice. Although this is true for di- and tri-nucleotides, for which both within and between-population heterozygosity estimates are equivalent after rarefaction, tetra-nucleotides remain inconsistent even when we consider rarefied estimates of heterozygosity.

The most likely explanation for this discrepancy seems to be some form of bias in the choice of markers. We know that tetra-nucleotide markers included in the HGDP-CEPH panel show significantly higher diversity than the unbiased markers developed by Romero *et al.* (2009). The patterns observed in the data would be consistent with a scenario where the HGDP-CEPH tetra-nucleotides were selected for high diversity from a panel mostly composed by Eurasians. It is difficult to say whether di- and tri-nucleotides were selected from a broader panel (and thus there was little or no bias in their selection process) or whether they escaped the effect of bias because of their higher mutation rates (*i.e.*, most markers have a diversity well above the threshold for selection). In any case, the discrepancy between different microsatellites of different motif lengths indicates that microsatellites are probably not as immune to ascertainment bias as suggested in the past (Rogers and Jorde 1996; Bowcock *et al.* 1994; Harpending and Rogers 2000).

It is clear that the choice of metrics extracted from the markers is important, as some are more affected by bias than others are. In contrast to the heterozygosity-based measures, we found STRUCTURE clusters to be relatively robust to the bias affecting tetra-nucleotides. This result is consistent with simulation studies of microsatellites and STRs, showing that STRUCTURE is relatively robust against ascertainment bias (Haasl and Payseur 2011).

Average heterozygosity is a commonly computed quantity, often used to parameterize population genetic models (Zhivotovsky *et al.* 2003; Liu *et al.* 2006; Deshpande *et al.* 2009). However, when the markers have heterogeneous mutation rates, this quantity does not correspond to the heterozygosity of the average mutation rate (or to any other single mutation rate). An important contribution of our article is the development of a rarefaction approach to combine markers with different mutation rates in heterozygosity-based measures corresponding to a single mutation rate. This approach allows all markers to carry equal weight in determining the overall metric, and is a much more accurate approach than simply averaging estimates obtained for different types of markers. Our approach is model free with respect to the underlying demographic processes (*i.e.*, we do not explicitly reconstruct bottlenecks, migrations, etc.). It is important to note that to combine heterozygosities, we do not need to know the mutation rate of any of the types but only have an *a priori* classification scheme that allows us to group markers with relatively similar mutation rates. However, if the mutation rate of one of the marker types is known, rarefaction yields the mutation rate of the combined heterozygosities. This way of estimating mutation rates has the advantage to be relatively robust to the inclusion of markers with extreme values, a known weakness of the commonly used approach of scaling the variances (Zhivotovsky *et al.* 2003).

Although we have not performed a formal investigation of the number of makers needed for our approach to be stable, we estimate our rarefaction method to require at least 10, and ideally over 20 markers in each group to avoid artifacts while rescaling. In the case of microsatellites, we obviously need markers to be clean enough to comply with the SMM framework. We provide several tools to check such compliance.

The SMM model is the simplest and most widely used model of microsatellite evolution, but it may seem overly simplistic given the heterogeneous nature of human microsatellites (Chakraborty *et al.* 1997; Ellegren 2004). For example, the fact that the SMM model assumes symmetric mutation rates may seem to limit its validity for our method. However, our method uses only the pairwise difference in number of repeats between individuals and is, therefore, independent of any constant, directional bias in the mutations (toward higher or lower repeat counts). It is also possible to use our method with more complex models. In the appendix, we show how our method can be adapted to use mutation models with arbitrary length-dependent increment and decrement rates and for models in which multiple repeat units can sometimes be added or removed. The main conclusion from analyzing these models is that the scaling relation between rarefied and original distribution of repeat counts depends only on the ratio of the two mutation rates, and they can be used in the same way as the simple SMM model. Although in many cases, it is not possible to write the scaling relation in a closed form, it is straightforward to compute it numerically. For the case of constraining the SMM model to have repeat counts between one and *L*, we show in the appendix that the scaling relation is the same as in the standard SMM model.

What are the implications of the bias in HGDP-CEPH tetra-nucleotides for studies that have used these markers to study the effect of SNP ascertainment bias? Conrad *et al.* (2006) plotted within-population haplotype heterozygosity against microsatellite heterozygosity for different haplotype lengths. Although SNPs were found to have little relation to microsatellites, they found that points fall on a curve for haplotypes at least 20 kb long (see Figure 3 in Conrad *et al.* 2006). If we were to remove the tetra-nucleotide markers from this plot, the American data points would be shifted strongly left, the Asian data points slightly so, and the remaining populations would be essentially unchanged (Figure 6). Although the resulting pattern would be slightly less linear, the points would still fall on a curve (because heterozygosity of microsatellites and SNPs depend differently on the underlying gene genealogies, the relation between the two is not linear in general). The conclusion that sufficiently long haplotypes are effectively free of ascertainment bias is not strongly affected by the bias in the tetra-nucleotides. Sun *et al.* (2009) found a linear relation between microsatellite average-squared difference (ASD) and sequence divergence for individuals from different populations. The bias observed in the tetra-nucleotides has little effect on ASD (unpublished) and, therefore, does not change any of their conclusions in that article.

For models describing the expansion of anatomically modern humans out of Africa that have been parameterized using the average heterozygosity of all markers (*e.g.*, Ramachandran *et al.* 2005; Liu *et al.* 2006; Deshpande *et al.* 2009), we would not expect the biases discussed in this article to put their main conclusions into doubt. On the other hand, we would expect the actual estimates of most parameters to be affected. This is especially true of the timing of the expansion out of Africa, which is usually the most interesting quantity. In addition, the relatively flat *F*_ST_ pattern from tetra-nucleotide markers (Figure 3), as well as the weaker cline in within-population expected heterozygosity (Figure 6), would cause such models to overestimate gene flow (migration) and underestimate population bottlenecks during the expansion; the magnitude of these effects depends on the details of the models, but it is approximately proportional to the relative effect of the bias on *F*_ST_ and the trend in expected heterozygosity.

The HGDP-CEPH panel has been used as a blueprint for the development of large datasets based on microsatellites, and subsets of the markers used in the HGDP-CEPH have been adopted for investigating genetic diversity in Africa (Tishkoff *et al.* 2009), India (Rosenberg *et al.* 2006), Oceania (Friedlaender *et al.* 2008), and the Americas (Wang *et al.* 2007). A simple precaution for future users of these datasets would be to limit their analysis to di- and tri-nucleotides. Although tetra-nucleotides constitute the majority of the markers in the HGDP-CEPH panel, di- and tri-nucleotides together still provide 220 markers, a very large number in terms of population genetics. Furthermore, our analyses above show that these markers are enough to perform all the type of analytical approaches usually employed on this type of data and, indeed, drive the results for measures such as *H* and *F*_ST_ when the data are combined. However, as several of these datasets concentrate on geographic areas at the boundaries of the coverage from the HGDP-CEPH panel, we would recommend following the same steps adopted in this article to investigate the presence of biases and make sure that they are fully removed.

## Supplementary Material

Supporting Information

## Appendix

### The SMM Model with Arbitrary Jump Distributions

Consider an extension of the standard SMM model to arbitrary jump distributions, such that at each mutation the probability of a change of *k* repeats is *q_k_* (with *q*_0_ = 0). The standard SMM model corresponds to taking *q*_1_= *q*_-1_= 1/2, and *q_k_* = 0 otherwise. Using the characteristic function

$$H(\omega )=1-\mu +\mu \sum_{k=-\infty }^{\infty }q_{k}e^{ik\omega }$$

we can write the characteristic function $\hat{p}(\omega ,\mu )$ for the difference Δ in repeat count between two alleles as

$$\hat{p}(\omega ,\mu )=\langle H{\left(\omega \right)}^{t}H{\left(-\omega \right)}^{t}\rangle =g\left(H(\omega )H(-\omega )\right)$$

where *g*(*z*) is the generating function for the distribution of the TMRCA. Expanding to the first order in µ, we obtain

$$\hat{p}(\omega ,\mu )=g\left(1-2\mu G(\omega )\right)$$

where

$$G(\omega )=1-\sum_{k=1}^{\infty }\left(q_{k}+q_{-k})\cos(k\omega \right)$$

Hence, for any small mutation rate µ,

$$g(z)=\hat{p}\left(G^{\left(-1\right)}\left(\frac{1-z}{2\mu }\right),\mu \right)$$

where $G^{\left(-1\right)}$ denotes the inverse function of *G*. Thus, for mutation rate µ′, we express $\hat{p}(\omega ,\mu \prime )$ in terms of $\hat{p}(\omega ,\mu )$:

$$\hat{p}(\omega ,\mu \prime )=g(1-2\mu \prime G(\omega ))=\hat{p}(G^{\left(-1\right)}\left(\frac{\mu \prime }{\mu }G(\omega \right)),\mu )$$

Finally, we can use the inverse Fourier transform to obtain the distribution of Δ at mutation rate µ′ from $\hat{p}(\omega ,\mu \prime )$. As for the standard SMM model, the scaling relation depends only on ratio of the two mutation rates, which means that our scaling method works equally well for these models.

We also note that only the component of the jump distribution symmetric around *k* = 0 enters into the final expression. This is a consequence of the fact that we consider only differences in repeat count, and implies that the scaling relation is independent of any systematic increase or decrease in the repeat count.

If the jump distribution is fully symmetric, the scaling relation is exact. Otherwise, there is a small correction to the scaling relation, but it is still possible to express $\hat{p}(\omega ,\mu \prime )$ in terms of $\hat{p}(\omega ,\mu )$. However, when the mutation rates are small the correction is negligible.

### The *L*-allele Mutation Model

In this section, we consider the effect of limiting the SMM models to repeat counts in the range [1, *L*] (shifting the range with an arbitrary amount makes no difference to the analysis). At the ends of the interval, we introduce reflecting boundary conditions such that the stationary distribution is uniform over the range. For alleles with repeat count in the range [2, *L*-1], mutations occur independently each generation with probability µ, with equal probability of gaining or losing a single repeat unit. At repeat count one, only mutations leading to an increment are accepted, and the repeat count *L* only decrements are accepted. Starting from a distribution *x* at time zero, the distribution of *p* of alleles *t* generations later can be written on matrix form as

$$p={\left(1-\mu M\right)}^{t}x$$

where the matrix M has elements given by *M_i,i_* = 1, *M_i_*_-1,_*_i_* = *M_i_*_+1,_*_i_* = −1/2, and *M_i_*_,_*_j_* = 0 otherwise, except at the boundaries where *M*_1,1_ = *M_L_*_,_*_L_* = 1/2. Correspondingly, the joint distribution *P_ij_* for two individuals becomes (given that *M* is symmetric)

$$P={\left(1-\mu M\right)}^{t}D{\left(1-\mu M\right)}^{t}$$ (A1)

where *D* denotes the diagonal matrix with diagonal elements equal to *x*.

Because *M* is symmetric, all eigenvectors and eigenvalues are real-valued, and the eigenvectors form an orthonormal basis. Expanding *M* in the eigenvectors *u*_α_ and eigenvalues *λ*_α_, we have

$${\left(1-\mu M\right)}^{t}=\sum_{\alpha =1}^{L}{\left(1-\mu \lambda_{\alpha }\right)}^{t}u_{\alpha }u_{\alpha }^{T}$$

where the superscript *T* denotes transpose. Inserting this in the equation for *P* above, and multiplying from the left and right with $u_{\alpha }^{T}$ and $u_{\beta }$, respectively, gives

$${\hat{P}}_{\alpha ,\beta }=u_{\alpha }^{T}Pu_{\beta }={\left(1-\mu \lambda_{\alpha }\right)}^{t}{\left(1-\mu \lambda_{\beta }\right)}^{t}u_{\alpha }^{T}Du_{\beta }$$

If we now assume that the distribution of alleles in the MRCA of the two individuals is the stationary distribution (*i.e.*, the locus is old enough), this equation simplifies to

$${\hat{P}}_{\alpha \beta }=\frac{1}{L}{\left(1-\mu \lambda_{\alpha }\right)}^{2t}\delta_{\alpha \beta }$$

where $\delta_{\alpha \beta }$ is Kroenecker’s delta, which is equal to one if α=β and is zero otherwise, ${\hat{P}}_{\alpha \beta }$ is a diagonal matrix. If we now consider an arbitrary distribution of TMRCA with generating function *g*(*z*), as in the previous section, we obtain

$${\hat{P}}_{\alpha \beta }(\mu )=\langle \frac{1}{L}{\left(1-\mu \lambda_{\alpha }\right)}^{2t}\delta_{\alpha \beta }\rangle =\frac{1}{L}\;g\left({\left(1-\mu \lambda_{\alpha }\right)}^{2}\right)\delta_{\alpha \beta }$$

Again, the function *g*(*z*) is independent of the mutation rate. Thus, this equation suggests a scaling relation $\mu \lambda_{\alpha }=\mu^{\prime }\lambda_{\alpha^{\prime }}$ between the eigenvalues at mutation rate *µ* and *µ*’ for the same underlying distribution of TMRCA. The joint distribution of alleles for mutation rate µ′, *P*(µ′), can then written in terms of the diagonal elements ${\hat{P}}_{\alpha \alpha }$:

$$P(\mu \prime )=\sum_{\alpha \prime =1}^{L}{\hat{P}}_{\alpha \prime ,\alpha \prime }(\mu \prime )u_{\alpha \prime }u_{\alpha \prime }^{T}=\sum_{\alpha \prime =1}^{L}{\hat{P}}_{\alpha (\alpha \prime ),\alpha (\alpha \prime )}(\mu )u_{\alpha \prime }u_{\alpha \prime }^{T}$$

where $\alpha (\alpha^{\prime })$ denotes the value of α corresponding to α′ under the scaling relation. A problem with this view is that the scaled values of α may not be an integer. In this case we can use that the underlying function *g*(*z*) is smooth: hence, if we sort the eigenvalues in increasing order, we can estimate ${\hat{P}}_{\alpha \alpha }$ at non-integer α using interpolation.

For the *L*-SMM, the eigenvalues are

$$\lambda_{\alpha }=1-\cos\left(\pi \left(\alpha -1\right)/L\right)$$

Solving the scaling relation gives the correspondence

$$\frac{\pi \left(\alpha -1\right)}{L}=\arccos\left(1-\frac{\mu \prime }{\mu }\left[1-\cos\left(\frac{\pi \left(\alpha \prime -1\right)}{L}\right)\right]\right)$$

Identifying the angular frequency ω=π(α−1)/L, we see that this relation is identical to the scaling relation for the standard SMM model (see Equation 1). Indeed, since the homozygosity *F* is the sum of the diagonal of *P*, and this scalar is invariant under unitary matrix transformations, we can write *F* as $F=\sum_{\alpha =1}^{L}{\hat{P}}_{\alpha \alpha }$ which is the direct correspondence to Equation 2 with Δ = 0.

### SMM Models with Length-Dependent Mutation Rates

The analyses in the previous section can be extended to single-step mutation models (with finite number of states) where the probability of increment and decrement mutations can both have an arbitrary dependence on the number of repeats in the sequence. As in the preceding section, we will assume that the locus is old enough that the ancestral probability distribution is approximately equal to the stationary distribution (this is an assumption implicit in methods typically used in estimating length-dependent mutation rates). Our starting point is Equation A1 in the preceding section, where *M* is the (tri-diagonal) mutation matrix, and *D* the diagonal matrix corresponding to the stationary distribution. Here, *M* is generally not symmetric, but it is straightforward to verify that the matrix *MD* is (it follows directly from the condition for having a stationary distribution). Hence, we can use an approach similar to above using

$$\tilde{P}(\mu )=D^{-1/2}P(\mu )D^{-1/2}$$

Inserting Equation A1, we obtain

$$\begin{array}{c} \tilde{P}(\mu )=D^{-1/2}\langle {\left(1-\mu M\right)}^{t}D{\left(1-\mu M^{T}\right)}^{t}\rangle D^{-1/2}\\ =\langle {\left(1-\mu D^{-1/2}MD^{1/2}\right)}^{t}{\left(1-\mu D^{1/2}M^{T}D^{-1/2}\right)}^{t}\rangle \\ =\langle {\left(1-\mu \tilde{M}\right)}^{t}{\left(1-\mu {\tilde{M}}^{T}\right)}^{t}\rangle \end{array}$$

The matrix $\tilde{M}$ is symmetric, as

$$\tilde{M}=D^{-1/2}MD^{1/2}=D^{-1/2}MDD^{-1/2}$$

Hence, we can use the method from the previous section to relate $\tilde{P}(\mu )$ to the generating function *g*(*z*) of the TMRCA of the locus,

$${\hat{P}}_{\alpha \beta }(\mu )=u_{\alpha }^{T}\tilde{P}(\mu )u_{\beta }=g\left({\left(1-\mu \lambda_{\alpha }\right)}^{2}\right)\delta_{\alpha \beta }$$

where $\lambda_{\alpha }$ are the eigenvalues of $\tilde{M}$ (which are identical to those of *M*), and the value of ${\hat{P}}_{\alpha \alpha }(\mu \prime )$, for 0 < *µ*′ < *µ*, can again be found by interpolation from ${\hat{P}}_{\alpha \alpha }(\mu )$. Finally, the joint distribution of alleles at the lower mutation rate can be calculated using

$$P(\mu \prime )=\sum_{\alpha =1}^{L}{\hat{P}}_{\alpha \alpha }(\mu \prime )D^{1/2}u_{\alpha }u_{\alpha }^{T}D^{1/2}$$

where $u_{\alpha }$ are the eigenvectors of $\tilde{M}$.
