# Supplementary material for: Detecting and Removing Ascertainment Bias in Microsatellites from the HGDP-CEPH Panel
Source: G3 (Bethesda). 2011 Nov 1;1(6):479–88. doi: 10.1534/g3.111.001016 (PMC3276161; doi:10.1534/g3.111.001016)
Supplement: Supporting Information [file supp_1_6_479__index.html]

Supporting Information 

# Detecting and Removing Ascertainment Bias in Microsatellites from the HGDP-CEPH Panel

## Supporting Infomation for Eriksson and Manica, 2011

**Files in this Data Supplement:**

- Supporting Information - Figures S1 and S2, Files S1-S4, and Tables S1 and S2 (PDF, 560 KB)
- Figure S1 - Allelic richness within populations (rarefied down to eight individuals), as a function of distance from sub-Saharan Africa, for di-, tri- and tetra-nucleotides separately (PDF, 188 KB)
- Figure S2 - Classifying the repeat motif length of STRs (PDF, 212 KB)
- Table S1 - Number of consistent, cleaned, and rejected di-, tri- and tetra-nucleotides in the HGDP-CEPH dataset (PDF, 60 KB)
- Table S2 - HGDP-CEPH markers where our classification differs from Pemberton *et al.* (PDF, 80 KB)
- File S1 - Matlab script for analysing microsatellites (.zip, 4 KB)
- File S2 - Cleaned HGDP-CEPH data in Structure format (.zip, 1.5 MB)
- File S3 - Cleaned unbiased data in Structure format (.zip, 36 KB)
- File S4 - Repeat motif length and cleaning status for HGDP-CEPH markers (Microsoft Excel, .xls, 80 KB)
