# Supplementary material for: Detecting and Removing Ascertainment Bias in Microsatellites from the HGDP-CEPH Panel
Source: G3 (Bethesda). 2011 Nov 1;1(6):479–88. doi: 10.1534/g3.111.001016 (PMC3276161; doi:10.1534/g3.111.001016)
Supplement: Supporting Information [file supp_1.6.479_001016SI.pdf]

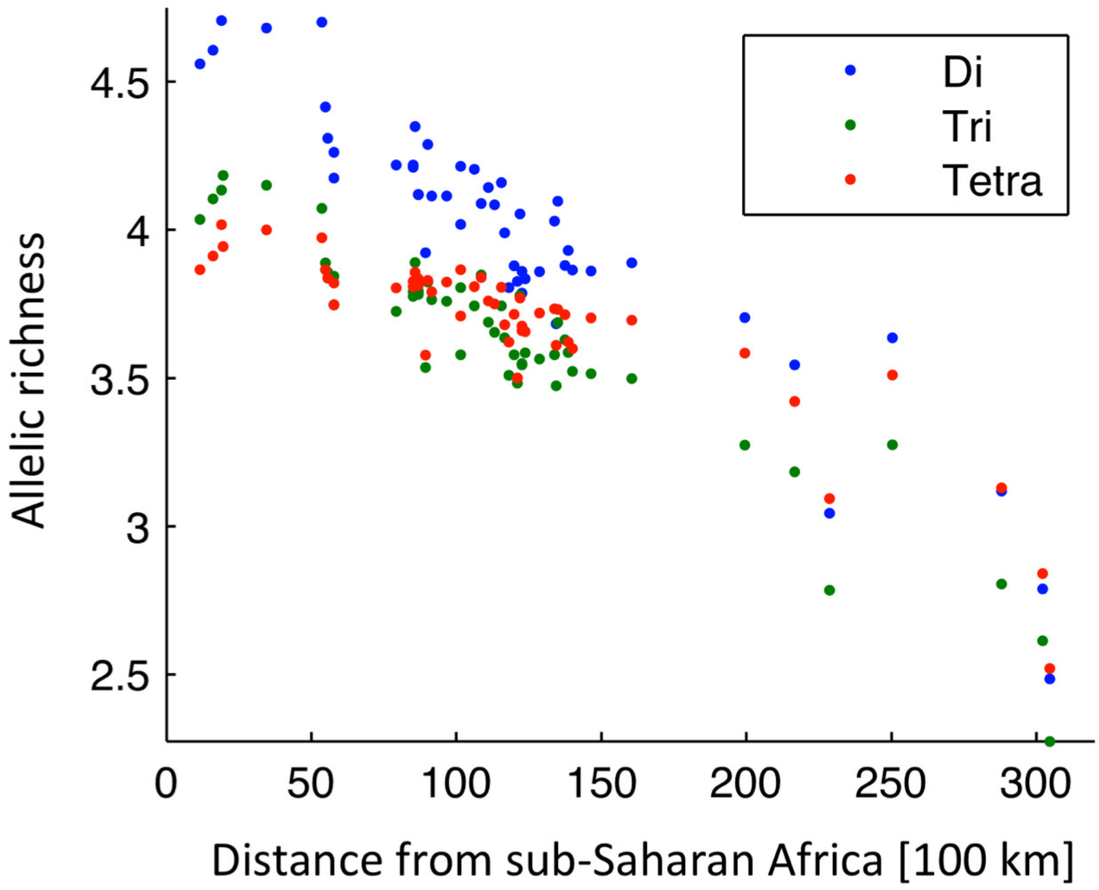


**Figure S1** Allelic richness within populations (rarefied down to eight individuals), as a function of distance from sub-Saharan Africa, for di-, tri- and tetra-nucleotides separately (see legend).

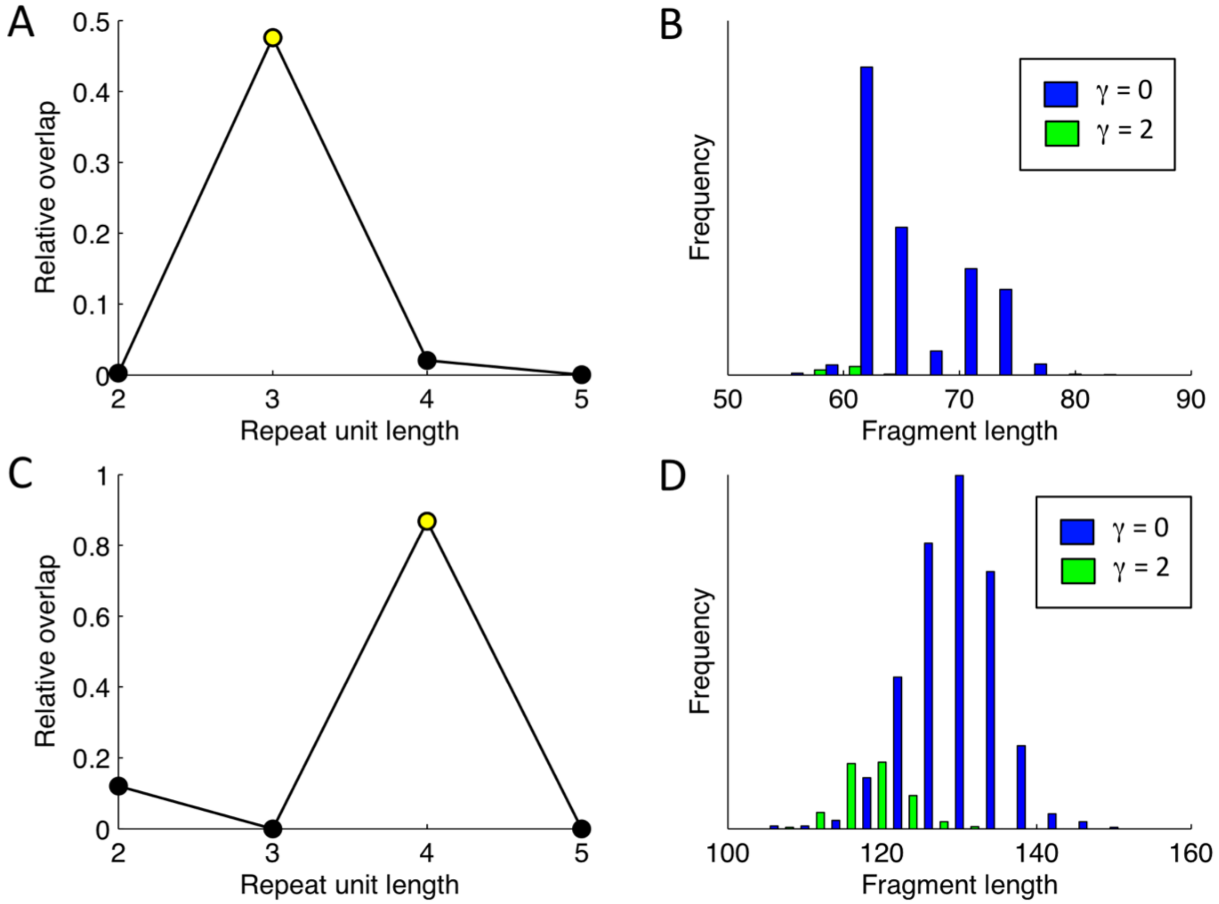


**Figure S2** Classifying the repeat motif length of STRs. (A,B) Example of a locus targeted for cleaning (misfit <5%). This is locus is clearly a tri locus with some noise, which could be cause by stuttering (or a point mutation). A: The relative overlap $\sum_i f_i f_{i+L} / \sum_i f_i^2$, where $f_i$ is the frequency of allele *i* in the sample, and *L* is the repeat unit length. Repeat length three has the best fit (yellow circle). B: The frequencies of alleles with different values of the remainder *g*. (C,D) Example of a locus targeted for removal. Panels C and D corresponds to panels A and B, respectively. This could be either a mix of di- and tetra-nucleotide repeats, or two shifted tetra-nucleotide sequences.

**Files S1-S4**

**Datasets**

Files S1-S4 are available for download at http://www.g3journal.org/lookup/suppl/doi:10.1534/g3.111.001016/-/DC1.

File S1: Matlab script for analysing microsatellites

File S2: Cleaned HGDP-CEPH data in Structure format

File S3: Cleaned unbiased data in Structure format

File S4: Repeat motif length and cleaning status for HGDP-CEPH markers

**Table S1 Number of consistent, cleaned, and rejected di-, tri- and tetra-nucleotides in the HGDP-CEPH dataset.**

| STR type | Consistent | Cleaned | Rejected | Total accepted |
|---|---|---|---|---|
| Dinucleotide | 47 | 7 | 1 | 54 |
| Trinucleotide | 166 | 0 | 6 | 166 |
| Tetranucleotide | 431 | 88 | 27 | 519 |
| Pentanucleotide | 7 | 3 | 0 | 10 |
| Total | 651 | 98 | 34 | 749 |

**Table S2 HGDP-CEPH markers where our classification differs from Pemberton *et al.* (T. J. PEMBERTON, C. I. SANDEFUR, M. JAKOBSSON, A. N. ROSENBERG, 2009 Sequence determinants of human microsatellite variability. *BMC Genomics* 10: 612).**

| Marker name | Pemberton et al. repeat length | Cleaned repeat length | Cleaning status |
|---|---|---|---|
| D3S2427 | 4 | 2 | Consistent |
| D16S764 | 2 | 4 | Consistent |
| D21S1446 | 4 | 2 | Consistent |
| D19S433 | 4 | 2 | Consistent |
| GTT035_13 | 3 | 4 | Rejected |
| TCTA023P_14 | 4 | 2 | Cleaned |
