# Supplementary material for: Detecting and Removing Ascertainment Bias in Microsatellites from the HGDP-CEPH Panel
Source: G3 (Bethesda). 2011 Nov 1;1(6):479–88. doi: 10.1534/g3.111.001016 (PMC3276161; doi:10.1534/g3.111.001016)
Supplement: Supporting Information [file supp_1.6.479_FigureS1.pdf]

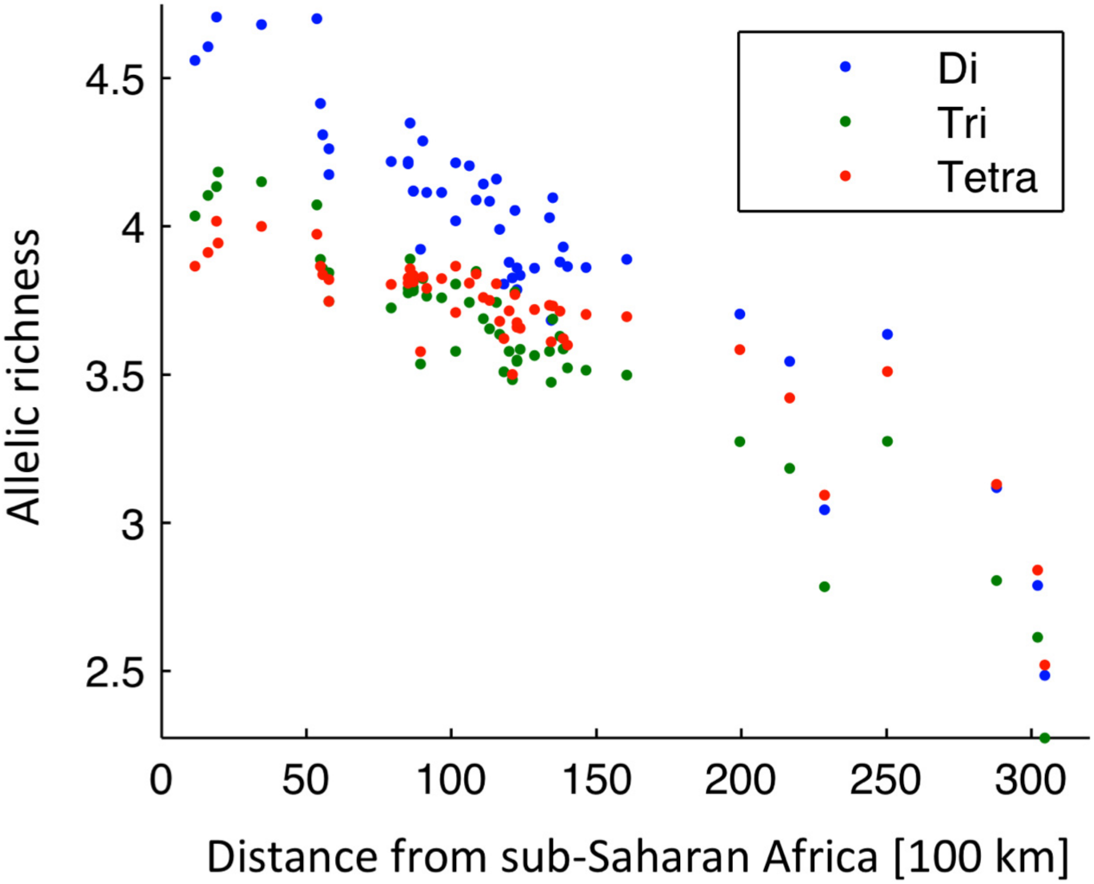

Figure S1 Allelic richness within populations (rarefied down to eight individuals), as a function of distance from sub-Saharan Africa, for di-, tri- and tetra-nucleotides separately (see legend).
