# Supplementary material for: Detecting and Removing Ascertainment Bias in Microsatellites from the HGDP-CEPH Panel
Source: G3 (Bethesda). 2011 Nov 1;1(6):479–88. doi: 10.1534/g3.111.001016 (PMC3276161; doi:10.1534/g3.111.001016)
Supplement: Supporting Information [file supp_1.6.479_FigureS2.pdf]

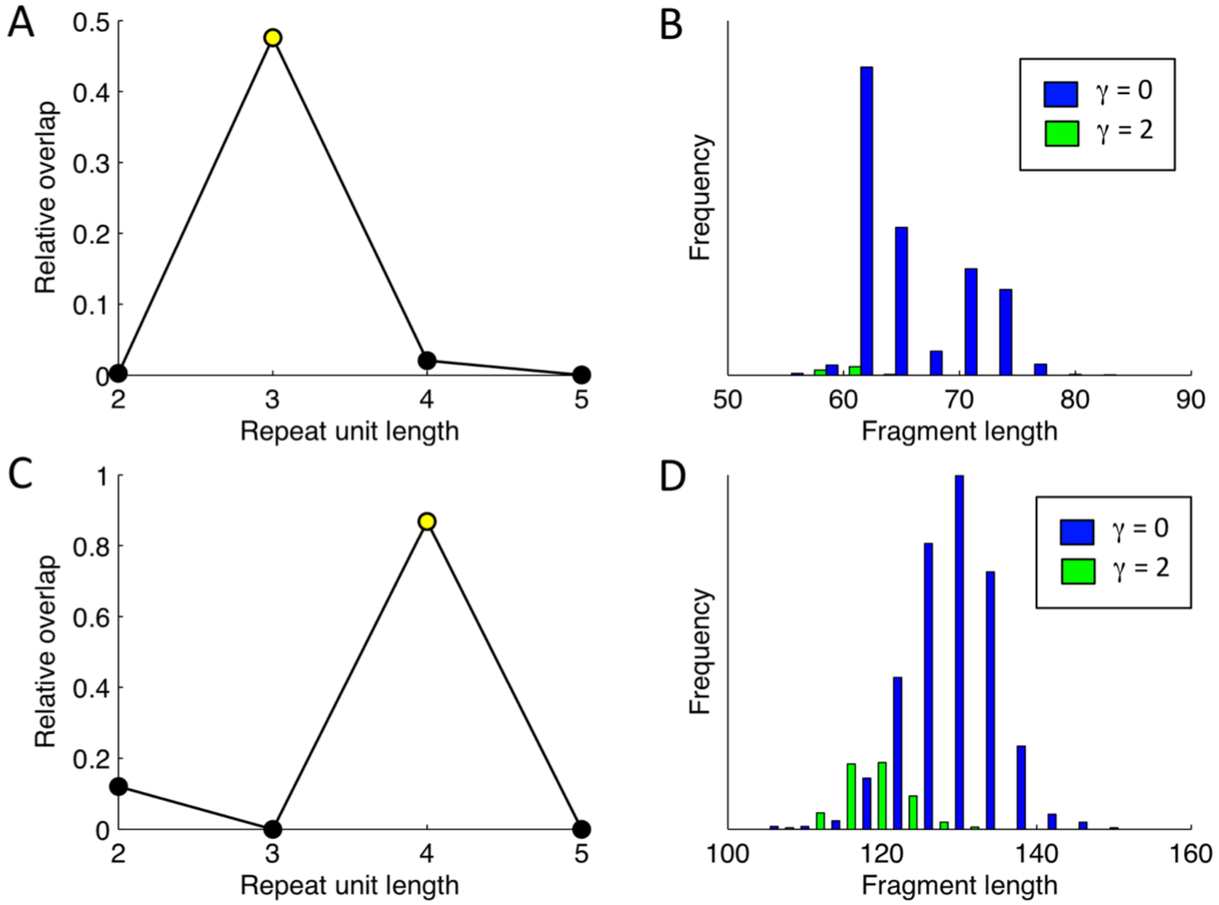

Figure S2 Classifying the repeat motif length of STRs. (A,B) Example of a locus targeted for cleaning (misfit <5%). This is locus is clearly a tri locus with some noise, which could be cause by stuttering (or a point mutation). A: The relative overlap $\sum_i f_i f_{i+L} / \sum_i f_i^2$, where f_i is the frequency of allele i in the sample, and L is the repeat unit length. Repeat length three has the best fit (yellow circle). B: The frequencies of alleles with different values of the remainder *g*. (C,D) Example of a locus targeted for removal. Panels C and D corresponds to panels A and B, respectively. This could be either a mix of di- and tetra-nucleotide repeats, or two shifted tetra-nucleotide sequences.
