# Supplementary material for: Detecting and Removing Ascertainment Bias in Microsatellites from the HGDP-CEPH Panel
Source: G3 (Bethesda). 2011 Nov 1;1(6):479–88. doi: 10.1534/g3.111.001016 (PMC3276161; doi:10.1534/g3.111.001016)
Supplement: Supporting Information [file supp_1.6.479_TableS1.pdf]

Table S1 Number of consistent, cleaned, and rejected di-, tri- and tetra-nucleotides in the HGDP-CEPH dataset.

STR type	Consistent	Cleaned	Rejected	Total accepted
Dinucleotide	47	7	1	54
Trinucleotide	166	0	6	166
Tetranucleotide	431	88	27	519
Pentanucleotide	7	3	0	10
Total	651	98	34	749
