# Supplementary material for: Detecting and Removing Ascertainment Bias in Microsatellites from the HGDP-CEPH Panel
Source: G3 (Bethesda). 2011 Nov 1;1(6):479–88. doi: 10.1534/g3.111.001016 (PMC3276161; doi:10.1534/g3.111.001016)
Supplement: Supporting Information [file supp_1.6.479_TableS2.pdf]

Table S2 HGDP-CEPH markers where our classification differs from Pemberton *et al.* (T. J. PEMBERTON, C. I. SANDEFUR, M. JAKOBSSON, A. N. ROSENBERG, 2009 Sequence determinants of human microsatellite variability. *BMC Genomics* 10: 612).

Marker name	Pemberton et al. repeat length	Cleaned repeat length	Cleaning status
D3S2427	4	2	Consistent
D16S764	2	4	Consistent
D21S1446	4	2	Consistent
D19S433	4	2	Consistent
GTT035_13	3	4	Rejected
TCTA023P_14	4	2	Cleaned
